# Automated Optic Disc Tilt Classification in Fundus Photographs Using Segmentation and the Elliptical Ratio: External Clinical Validation Study

**DOI:** 10.2196/86380

**Published:** 2026-07-02

**Authors:** Chae Yeon Lim, Jaeryung Kim, Joonhyoung Kim, Chaeyeon Lee, Euido Song, Myung Jin Chung, Sei Yeul Oh, Taeyoung Kim, Kyung Ah Park

**Affiliations:** 1 Department of Medical Device Management and Research, Samsung Advanced Institute for Health Sciences and Technology (SAIHST), Sungkyunkwan University, Seoul, Republic of Korea; 2 Medical AI Research Center, Samsung Medical Center, Seoul, Republic of Korea; 3 Seoul National University, Seoul, Republic of Korea; 4 OGQ, Seoul, Republic of Korea; 5 Department of Ophthalmology, Sungkyunkwan University School of Medicine, Samsung Medical Center, 81 Irwon-Ro, Gangnam-gu, Seoul, 06351, Republic of Korea; 6 Department of Data Convergence and Future Medicine, Sungkyunkwan University School of Medicine, Seoul, Republic of Korea; 7 Department of Radiology and Medical AI Research Center, Samsung Medical Center, Seoul, Republic of Korea

**Keywords:** digital health, myopia, optic disc, optic disc tilt, fundus photography, segmentation, artificial intelligence, AI, ophthalmologists, telemedicine, ophthalmic image analysis, computer-assisted diagnosis, retinal imaging

## Abstract

**Background:**

Optic disc tilt is a morphological change in myopic eyes that complicates clinical interpretation and artificial intelligence (AI)–based analysis of fundus images. Accurate detection of optic disc tilt is necessary to avoid misinterpretation of disc morphology and enhance diagnostic reliability across different disease types.

**Objective:**

This study developed and externally validated an end-to-end AI-based pipeline for optic disc segmentation and quantitative tilt classification in color fundus photographs (CFPs), offering an objective alternative to manual segmentation and subjective clinical assessments.

**Methods:**

We trained a nnU-Net–based optic disc segmentation model on the Standardized Multi-Channel Dataset for Glaucoma (SMDG; n=3103 CFPs) and externally validated it on the Samsung Medical Center (SMC) dataset (n=2448 CFPs from n=1370 patients). Model generalizability was assessed using both a fixed 80:20 random split and 5-fold cross-validation. Tilt was classified using the ratio of the long-axis diameter to the short-axis diameter, with a ratio of ≥1.3 indicating tilt. Segmentation performance was evaluated using the Dice similarity coefficient, intersection over union, and pixel accuracy on the SMDG dataset and using the clinical acceptance rate determined by 2 independent ophthalmologists on the external SMC dataset.

**Results:**

Using the SMDG dataset, nnU-Net achieved consistently high performance, with mean Dice similarity coefficients of 0.956 (SD 0.042) across 5-fold cross-validation and 0.961 (SD 0.055) for the best-performing single-fold model across 8 datasets. On the SMC dataset, 2 independent expert reviews yielded mean clinical acceptance rates of 98.61% and 98.86% across disease types, with acceptance rates ranging from 81.63% and 93.88% for edema to 99.59% and 99.17% for pallor, respectively. Tilt was detected in 7.5% (186/2448) of images, with rates of 9.7% (118/1215) for normal images, 3.9% (35/894) for glaucoma, 7.8% (19/241) for pallor, and 14.2% (14/98) for edema. Segmentation errors were observed in 1.39% (34/2448) and 1.14% (28/2448) of cases by the 2 reviewers, mainly due to edema-related swelling, peripapillary atrophy, and vessel confusion.

**Conclusions:**

Our pipeline provides objective and reproducible detection of optic disc tilt in CFPs, with strong generalizability to clinical images. By replacing manual segmentation and subjective assessments, the pipeline supports tilt-aware AI diagnostics and scalable screening for myopia-related conditions, with future refinements needed to address edema-related challenges.

## Introduction

Myopia has become a global epidemic, with prevalence among children and adolescents increasing from 24.32% in 1990 to 35.81% in 2023 and projected to reach 39.80% by 2050 [1]. This trend is particularly pronounced in East Asian countries, where myopia prevalence exceeds 80% to 90% among young adults, with high myopia (≥−6.00 diopters) affecting an increasing proportion of the population [2]. High myopia is associated with various structural ocular changes, with optic disc tilt representing one of the most common morphological alterations. A myopic tilted disc appears as an oval-shaped and obliquely rotated optic nerve head resulting from mechanical stress during axial elongation [3]. The approximate prevalence of optic disc tilt ranges from 10% to 80%, with higher rates consistently observed in populations with high myopia [4].

Optic disc tilt poses significant clinical challenges for ophthalmologists, as it complicates the interpretation of optic disc morphology and the detection of pathological changes, such as glaucoma and papilledema. Alterations to the retinal nerve fiber layer and ganglion cell-inner plexiform layer, combined with changes in optic disc appearance, make it extremely difficult to differentiate between physiological variations associated with myopia and pathological changes requiring clinical intervention [5,6]. Even experienced specialists encounter substantial difficulties accurately assessing optic disc abnormalities in the presence of severe optic disc tilt. This challenge is further exacerbated in the context of artificial intelligence (AI), as deep learning–based optic disc classification systems have been shown to exhibit significantly reduced accuracy, sensitivity, and specificity when distinguishing among normal optic discs, glaucomatous changes, papilledema, and optic disc pallor in the presence of optic disc tilt [7]. Despite extensive research in AI-based fundus image analysis, optic disc tilt has not been explicitly considered as a preanalysis factor in most existing systems [8,9]. Our previous work explored the application of deep learning algorithms in computer-aided recognition of myopic tilted optic discs in fundus photography, but this approach primarily relied on subjective clinical assessments for ground-truth labeling and focused on classification rather than on robust quantitative characterization of optic disc morphology [10]. This limited consideration of optic disc tilt as an essential preanalysis factor represents a significant limitation, as accurate optic disc segmentation is fundamental to subsequent morphological analysis and the detection of abnormalities [11]. Therefore, the development of robust segmentation methods that can effectively handle optic disc tilt is needed to create reliable AI-assisted diagnostic tools.

The ultimate goal of developing AI-assisted methods for automated optic disc analysis is to create a comprehensive diagnostic framework that can accurately identify optic disc abnormalities regardless of the degree of optic disc tilt present. To address the limitations of existing approaches, this study introduces a novel AI-assisted pipeline designed for the automated detection and quantitative characterization of optic disc tilt in color fundus photographs (CFPs). By moving beyond subjective assessments and binary classification, the pipeline aims to provide objective and reproducible measurements of optic disc morphology, thereby establishing a crucial foundational step toward more robust, tilt-aware, and clinically applicable AI tools for the diagnosis of various optic disc abnormalities. This advancement is critical for improving the reliability of AI diagnostics in ophthalmology, particularly in the context of high myopia.

## Methods

### Ethical Considerations

This study was approved by the institutional review board of Samsung Medical Center (SMC; 2024-05-131) and followed the principles of the Declaration of Helsinki. All data were collected retrospectively from the institution’s data management team, and all direct identifiers, such as names and resident registration numbers, were fully deidentified through a standardized deidentification process.

### Study Design

**Figure 1.**
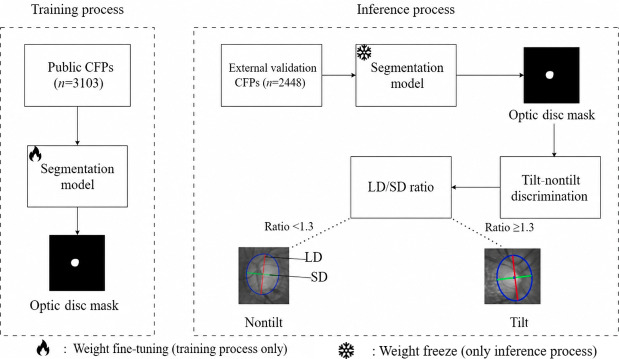
Overview of the optic disc segmentation and tilt classification pipeline. The segmentation model was pretrained using public color fundus photographs (CFPs) and applied to an external validation dataset. Tilt classification was performed based on the ratio of the optic disc’s long and short axes. LD: long diameter; SD: short diameter.

This study proposed a 2-stage, end-to-end pipeline to automatically determine optic disc tilt from CFPs, as illustrated in Figure 1. The overall framework comprises 2 sequential stages: optic disc segmentation and tilt detection based on the ratio of the optic disc’s long and short axes.

In the segmentation process, a model was trained and validated on 3103 CFPs from the public Standardized Multi-Channel Dataset for Glaucoma (SMDG) [12], which contains optic disc annotations. The model was optimized to extract a binary segmentation mask representing the optic disc region. In the subsequent tilt detection step, the pretrained segmentation model was used to infer optic disc regions from the external validation dataset, which comprised 2448 CFPs acquired from SMC. For each predicted optic disc mask, the long diameter (LD) and short diameter (SD) were measured relative to the geometric center of the mask (see equations below) [13]. The tilt ratio was defined as the LD divided by the SD, and a cutoff of 1.3 was applied to classify the optic disc as tilted (LD:SD ratio ≥1.3) or nontilted (LD:SD ratio <1.3) [14,15].

### Data Acquisition

The data used in this study were collected retrospectively from CFPs taken at the SMC Department of Ophthalmology between December 2007 and October 2021. A total of 1370 patients with 2448 CFPs were included in this study. The number of patients in each disease class was as follows: 514 healthy patients, 617 with glaucoma, 173 with pallor, and 66 with edema. The actual number of images in each disease class was as follows: 1215 normal, 894 glaucoma, 241 pallor, and 98 edema images. Images were excluded if their resolution was too low to allow clinical evaluation of the optic disc and related structures or if they showed severe artifacts, such as poor focus, strong light reflection, or motion blurring. The demographic and clinical characteristics of the study population are summarized in Table 1. The mean age was lowest in the normal group. The proportion of male patients ranged from 38% (n=66) to 55% (n=617), and the spherical equivalent refraction showed a tendency toward myopia in all groups.

**Table 1.** Demographic and clinical characteristics of patients in the Samsung Medical Center dataset (N=1370).

Characteristics	Normal group (n=514)	Patients with glaucoma (n=617)	Patients with pallor (n=173)	Patients with edema (n=66)
Age (years), mean (SD)	11.1 (11.8)	58.6 (12.8)	41.7 (19.9)	37.6 (15.7)
Male patients, %	245 (48)	339 (55)	90 (52)	25 (38)
Spherical equivalent refraction, mean (SD)	−2.24 (2.98)	−2.37 (2.88)	−1.49 (2.81)	−1.25 (2.08)

### Model Development and External Validation

#### Segmentation Model Development

Accurate optic disc segmentation is crucial for computing the LD and SD used to assess disc tilt. We used nnU-Net [16], a convolutional neural network (CNN)–based architecture known for its strong performance in biomedical image segmentation [17], and applied preprocessing steps to enhance training efficiency and accuracy [18,19]. The SMDG, consisting of 3103 CFPs, was used for model training and evaluation. This dataset integrates 6 public datasets sourced from 5 countries (Table 2): REFUGE (Guangzhou, China) [20], PAPILA (Murcia, Spain) [21], ORIGA (Southwestern Singapore) [22], G1020 (Kaiserslautern, Germany) [23], DRISHTI-GS1 (Madurai, India) [24], and CRFO-v4 (country of origin not specified) [25].

**Table 2.** Summary of Standardized Multi-Channel Dataset for Glaucoma with optic disc annotations.

Datasets	Nonglaucoma (n=2310), n (%)	Glaucoma (n=725), n (%)	Glaucoma suspect (n=68), n (%)
CRFO-v4	20 (0.87)	25 (3.4)	0 (0)
REFUGE-VALIDATION	360 (15.6)	40 (5.5)	0 (0)
REFUGE-TRAIN	360 (15.6)	40 (5.5)	0 (0)
PAPILA	333 (14.4)	87 (12)	68 (100)
ORIGA-light	482 (20.9)	168 (23.2)	0 (0)
G1020	724 (31.3)	295 (40.7)	0 (0)
DRISHTI-GS1-TEST	13 (0.56)	38 (5.3)	0 (0)
DRISHTI-GS1-TRAIN	18 (0.77)	32 (4.4)	0 (0)

All images were preprocessed by resizing them to 512×512 pixels to standardize spatial resolution across heterogeneous datasets, and black borders were removed via contour-based detection, which identified the largest connected foreground region and cropped images to its bounding extent. To minimize vessel interference and mitigate variations in illumination across different acquisition environments, all images were converted to gray scale [26,27] and their pixel values were normalized to a range between 0 and 1 [28]. Additionally, nnU-Net’s built-in preprocessing pipeline was applied, which includes automatic intensity normalization and patch-based training configuration derived from dataset fingerprint analysis. The preprocessed CFPs were used as input to the nnU-Net model, with the corresponding optic disc masks serving as ground truth. The SMDG dataset (N=3103) was divided into training (n=2483, 80%) and test (n=620, 20%) sets using random sampling (single-fold model; Table 3) [29]. To provide a more rigorous estimate of model generalizability, 5-fold cross-validation was additionally performed on the SMDG dataset, and the best-performing fold model was selected for all subsequent tilt analyses. Training was carried out using the PyTorch framework with the following hyperparameters: an initial learning rate of 1e–2, weight decay of 3e–5, batch size of 40, and 1000 training epochs. The Adam optimizer was used, and all experiments were run on an NVIDIA RTX A6000 GPU (NVIDIA Corp).

**Table 3.** Summary of the random sampling–based distribution of images in the training and test sets (N=3103).

Datasets	Training set (n=2483), n (%)	Test set (n=620), n (%)
CRFO-v4	35 (1.5)	10 (1.7)
REFUGE-VALIDATION	319 (12.8)	81 (13.0)
REFUGE-TRAIN	316 (12.7)	84 (13.5)
PAPILA	400 (16.1)	88 (14.3)
ORIGA-light	514 (20.7)	136 (21.9)
G1020	814 (32.8)	205 (33.0)
DRISHTI-GS1-TEST	43 (1.7)	8 (1.3)
DRISHTI-GS1-TRAIN	42 (1.7)	8 (1.3)

#### Related Work

Accurate segmentation of the optic disc is a prerequisite for the automated analysis of various ocular pathologies. With the advent of deep learning, numerous CNN architectures have been proposed to enhance segmentation performance. Table 4 summarizes previous methods for optic disc segmentation.

**Table 4.** Summary of deep learning–based optic disc segmentation methods and their performance in previous studies.

References	Datasets	Models	Dice similarity coefficient (%)	Intersection over union (%)
[30]	DRISHTI-GS1	U-Net	95.5	90.62
[31]	REFUGE and RIM-ONE	Inception V3	80.0	70.0
[32]	RIM-ONE and DRISHTI-GS1	Ensemble (U-Net and MobileNetV2)	92.0	—^a^
[33]	REFUGE and DRISHTI-GS1	Ensemble (DenseNet201 and ResNet18)	88.0	—
[34]	RIM-ONE, ORIGA, DRISHTI-GS-1, ACRIMA, and REFUGE	Ensemble (DeepLab v3 and MobileNet)	91.7	84.9
[35]	DRISHTI-GS1 and RIM	Ensemble convolutional neural network	93.7	88.5
[36]	DRISHTI-GS1 and REFUGE	W-Net	97.8	95.6

a Corresponding metric was not reported in the original study.

Deep learning architectures for optic disc segmentation and glaucoma diagnosis have evolved significantly. Initial approaches primarily used standard CNN backbones. For instance, Juneja et al [30] applied a modified U-Net architecture for the sequential segmentation of the optic disc and cup. Similarly, Neto et al [31] used a U-Net framework integrated with the Inception V3 model to derive the vertical cup-to-disc ratio (CDR) as a key diagnostic biomarker. To enhance robustness and generalization, more recent studies have shifted toward ensemble learning and transfer learning strategies. Civit-Masot et al [32] proposed a pipeline combining a generalized U-Net for segmentation with a pretrained MobileNetV2 for classification. This trend continued with Agrawal et al [33], who implemented a dual-CNN segmentation approach using Contrast Limited Adaptive Histogram Equalization–enhanced inputs, which subsequently fed into an ensemble of DenseNet201 and ResNet18 for glaucoma detection. Furthermore, Sreng et al [34] demonstrated the efficacy of integrating DeepLab v3+ with MobileNet features, using support vector machines for the final classification.

More recently, Zilly et al [35] proposed an ensemble CNN architecture based on entropy sampling and boosting, achieving a Dice similarity coefficient (DSC) of 93.7% across the DRISHTI-GS and RIM-ONE datasets. Building on this trajectory, Tang et al [36] introduced W-Net, a boundary-aware cascade network that achieved DSCs of 97.8% on DRISHTI-GS1 and REFUGE, representing one of the highest reported performances in optic disc segmentation to date. Existing studies primarily focus on pixel-level metrics or vertical CDR for glaucoma screening, often lacking validation across diverse pathologies such as edema or pallor. Crucially, they overlook geometric structural changes such as optic disc tilt. Notably, although several prior methods have achieved DSCs exceeding 0.95, these results are typically obtained by training and evaluating models within a single homogeneous dataset. In contrast, our approach was trained and evaluated across 8 heterogeneous subdatasets spanning 5 countries and multiple acquisition devices, where robust generalization across domain shift, rather than peak within-dataset performance, was the primary objective. Furthermore, our goal extends beyond segmentation accuracy itself. We aimed to derive objective tilt-related morphologic measurements via the LD:SD ratio across a broader range of optic disc appearances, including pallor and edema, which are not typically addressed in prior glaucoma-centric models. However, we acknowledge that complex conditions such as disc edema represent challenging cases in which segmentation-based tilt assessment should be interpreted cautiously and within the broader clinical context.

#### Tilt Detection on External Validation Dataset

The proposed method assesses optic disc tilt based on quantitative measurements derived from segmentation masks. On the external validation dataset (ie, the SMC dataset), optic disc masks were automatically obtained using a pretrained segmentation model.

Tilt was determined through geometric computation. Two orthogonal axes, the LD and SD, were defined by lines passing through the center of the optic disc mask. Line LD was defined as the longest chord between any 2 boundary points passing through the center, with LD denoting its length (Eq. 1). Line SD was defined as the chord perpendicular to line LD passing through the same center, with SD denoting its length (Eq. 2).



(1)
$$\begin{array}{rl} LD & =\max_{i,j}\sqrt{(x_{i}-x_{j}{)}^{2}+(y_{i}-y_{j}{)}^{2}},\\ \;\text{where line }LD\text{ passes through }(x_{c},y_{c}) \end{array}$$

(2)
$$\begin{array}{rl} SD & =\sqrt{(x_{k}-x_{l}{)}^{2}+(y_{k}-y_{l}{)}^{2}},\\ \;\text{where line }SD⊥\text{ line }LD\\ \text{ and passes through }(x_{c},y_{c}) \end{array}$$

Here, $\left(x_{i},y_{i}\right),\;\left(x_{j},y_{j}\right),\;\left(x_{k},y_{k}\right),\;\left(x_{l},y_{l}\right)\in B$, where B denotes the set of pixel coordinates along the boundary of the optic disc mask and $x_{c},y_{c})$ denotes the center of the optic disc.

### Performance Measurement

To quantitatively evaluate segmentation performance, we used 3 metrics: the DSC (Eq. 3) [37], intersection over union (Eq. 4) [38], and pixel accuracy (Eq. 5). These metrics are defined as follows:



(3)
$$DSC=\frac{2\left|P\cap G\right|}{\left|P\right|+\left|G\right|},$$

(4)
$$IoU=\frac{\left|P\cap G\right|}{\left|P\cup G\right|}$$

(5)
$$\text{Pixel Accuracy}=\frac{TP+TN}{TP+FP+TN+FN}$$

Where P and G denote the predicted segmentation mask and the ground-truth mask, respectively, and TP, TN, FP, and FN represent true-positive, true-negative, false-positive, and false-negative pixels, respectively. The 3 metrics range from 0 to 1, with values closer to 1 indicating higher similarity to the ground truth mask. These quantitative evaluations were performed using the SMDG, which provides ground-truth annotations. In contrast, the external validation dataset (ie, the SMC dataset) lacked annotation masks. To address this, we used the clinical acceptance rate as a human-based alternative for validating segmentation performance in the absence of ground-truth annotations. For the external validation dataset, segmentation masks were obtained by applying the pretrained segmentation model. The resulting masks were then evaluated independently by 2 ophthalmologists, and clinical acceptance rates were calculated separately for each reviewer to assess agreement with clinical judgment [39].

## Results

### Segmentation Performance Using the SMDG

To identify the most suitable backbone for optic disc segmentation, we compared the performance of 4 representative 2D CNN–based architectures: U-Net [40], Attention U-Net [41], TransU-Net [42], and nnU-Net. All models were trained and evaluated under the same experimental conditions using the SMDG.

Table 5 summarizes the comparative performance of the models. Among the models, nnU-Net achieved the highest DSC and was selected as the backbone for the proposed pipeline. The results of 5-fold cross-validation for the selected nnU-Net model are summarized in Table 6. Performance was highly consistent across all 5 folds (DSC 0.956‐0.957), indicating stable generalizability without sensitivity to any particular data partition. Table 7 presents the per-dataset segmentation performance of both the single-fold model with the highest DSC among the 5 folds and the 5-fold homogeneous ensemble models across the 8 SMDG subdatasets. The single-fold model recorded a DSC >0.94, intersection over union >0.90, and pixel accuracy >0.997 across all datasets. The ensemble showed comparable but marginally lower performance than the single-fold model across most subdatasets. Given that accurate segmentation is a critical prerequisite for reliable tilt quantification, the single-fold model with the highest DSC among the 5 folds was selected for all subsequent tilt analyses.

**Figure 2.**
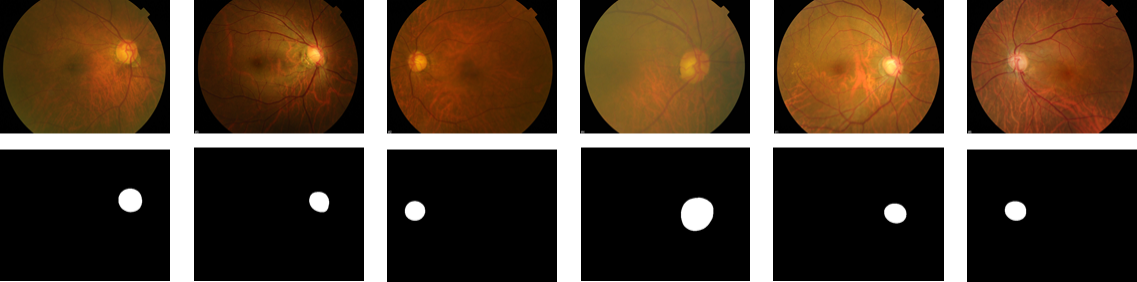
Example color fundus photographs (top) and corresponding predicted optic disc segmentation masks (bottom).

Figure 2 presents visual examples of the segmentation results. The top row shows original CFPs, and the bottom row shows the corresponding predicted masks.

**Table 5.** Comparison of 2D convolutional neural network models for optic disc segmentation.

Models	Dice similarity coefficient, mean (SD)	Intersection over union, mean (SD)	Pixel accuracy, mean (SD)
Attention U-Net	0.903 (0.102)	0.835 (0.121)	0.996 (0.0042)
Trans U-Net	0.905 (0.088)	0.836 (0.113)	0.998 (0.0026)
U-Net	0.933 (0.055)	0.882 (0.077)	0.997 (0.0015)
nnU-Net	0.961 (0.055)	0.927 (0.057)	0.996 (0.0011)

**Table 6.** Five-fold cross-validation performance of nnU-Net on the Standardized Multi-Channel Dataset for Glaucoma (N=3103).

Folds	Dice similarity coefficient, mean (SD)	Intersection over union, mean (SD)	Pixel accuracy, mean (SD)
Fold 1	0.957 (0.040)	0.919 (0.052)	0.998 (0.0011)
Fold 2	0.956 (0.042)	0.918 (0.035)	0.998 (0.0012)
Fold 3	0.956 (0.041)	0.918 (0.041)	0.998 (0.0012)
Fold 4	0.956 (0.043)	0.918 (0.042)	0.998 (0.0010)
Fold 5	0.956 (0.042)	0.918 (0.061)	0.998 (0.0012)
Mean	0.956 (0.042)	0.918 (0.046)	0.998 (0.0012)

**Table 7.** Segmentation performance on the Standardized Multi-Channel Dataset for Glaucoma.

Datasets	Single-fold model			Ensemble model (5-fold)		
	Dice similarity coefficient, mean (SD)	Intersection over union, mean (SD)	Pixel accuracy, mean (SD)	Dice similarity coefficient, mean (SD)	Intersection over union, mean (SD)	Pixel accuracy, mean (SD)
CRFO-v4 (n=10)	*0.973 (0.010)^a^*	*0.942 (0.019)*	0.998 (0.0003)	0.964 (0.013)	0.932 (0.020)	0.998 (0.0005)
REFUGE-VALIDATION (n=81)	*0.967 (0.020)*	*0.936 (0.036)*	0.998 (0.0005)	0.961 (0.018)	0.926 (0.033)	0.997 (0.0005)
REFUGE-TRAIN (n=84)	*0.968 (0.021)*	*0.939 (0.039)*	0.998 (0.0006)	0.959 (0.022)	0.922 (0.038)	0.998 (0.0006)
PAPILA (n=88)	*0.965 (0.020)*	*0.934 (0.037)*	0.997 (0.0010)	0.963 (0.022)	0.930 (0.040)	0.995 (0.0011)
ORIGA-light (n=136)	*0.968 (0.022)*	*0.939 (0.040)*	0.998 (0.0007)	0.965 (0.030)	0.933 (0.049)	0.998 (0.0009)
G1020 (n=205)	*0.948 (0.060)*	*0.905 (0.079)*	0.998 (0.0010)	0.945 (0.061)	0.901 (0.080)	0.997 (0.0014)
DRISHTI-GS1-TEST (n=8)	0.977 (0.010)	0.955 (0.019)	0.998 (0.0009)	*0.978 (0.008)*	*0.957 (0.015)*	0.998 (0.0004)
DRISHTI-GS1-TRAIN (n=8)	*0.969 (0.017)*	*0.940 (0.033)*	0.998 (0.0008)	0.959 (0.033)	0.924 (0.059)	0.998 (0.0018)

a Italics indicate the best-performing model for each dataset.

### Optic Disc Tilt Classification Performance on the SMDG Dataset

The performance of the optic disc tilt classification model was evaluated using the SMDG test set (n=620). Ground-truth labels were defined using the standard LD:SD ratio threshold of ≥ 1.3. At the clinically established threshold of an LD:SD ratio of ≥1.3, the model achieved an accuracy of 96.45% (598/620), a specificity of 98.27% (568/578), and a sensitivity of 71.43% (30/42). To characterize the distribution of misclassifications near the decision boundary, additional thresholds of ≥1.28 and ≥1.26 were evaluated (Table 8). The progressive recovery of sensitivity at lower thresholds indicates that misclassifications were concentrated in geometrically borderline cases. In these instances, the predicted LD:SD ratios were marginally lower (eg, 1.25-1.29) than the threshold, while the ground-truth ratios were slightly above 1.3. This pattern suggests that the misclassifications primarily reflect the limited representation of borderline tilt cases in the current dataset rather than a fundamental failure of the segmentation model itself, as the underlying segmentation maintained morphologic measurements that were very close to the ground truth. This dataset-driven limitation is expected to be mitigated in future work through the inclusion of additional borderline cases obtained through ongoing clinical data collection.

**Table 8.** Performance of optic disc tilt classification on the Standardized Multi-Channel Dataset for Glaucoma test set (N=620).

LD:SD ratio threshold	Predicted tilt	Predicted nontilt	Accuracy
≥1.30			96.45%
Actual tilt	30	12	
Actual nontilt	10	568	
≥1.28			95.30%
Actual tilt	32	10	
Actual nontilt	19	559	
≥1.26			94.19%
Actual tilt	35	7	
Actual nontilt	29	549	

### Expert Review of AI Segmentations on the SMC Dataset

Two ophthalmologists independently reviewed each of the 2448 CFPs in the SMC dataset. To facilitate visual assessment, each CFP was displayed alongside its predicted segmentation mask, enabling the experts to determine clinical acceptability (1=“acceptable” and 0=“unacceptable”). The clinical acceptance rate was computed separately for each reviewer.

Both experts reported consistently high clinical acceptance rates across the normal (n=1209, 99.35% and n=1198, 98.61%), glaucoma (n=885, 99.11% and n=891, 99.67%), and pallor (n=240, 99.59% and n=239, 99.17%) groups, indicating reliable segmentation performance in these categories (Table 9). Acceptance rates were comparatively lower in the edema group (n=80, 82.63% and n=92, 93.88%), although most cases were still considered clinically acceptable by both reviewers. Across the entire dataset of 2448 images, the model achieved mean clinical acceptance rates of 98.61% (n=2414; expert 1) and 98.86% (n=2420; expert 2), with only 34 (1.39%) and 28 (1.14%) cases, respectively, assessed as clinically unacceptable.

**Table 9.** Summary of the clinical acceptance rate for optic disc segmentation for each disease type in the Samsung Medical Center external validation dataset.

Datasets	Normal (n=1215)	Glaucoma (n=894)	Pallor (n=241)	Edema (n=98)	Total (N=2448)
Expert 1, n (%)	1209 (99.51)	885 (98.99)	240 (99.59)	80 (81.63)	2414 (98.61)
Expert 2, n (%)	1198 (98.61)	891 (99.67)	239 (99.17)	92 (93.88)	2420 (98.86)

### Tilt Evaluation by Disease Type

**Figure 3.**
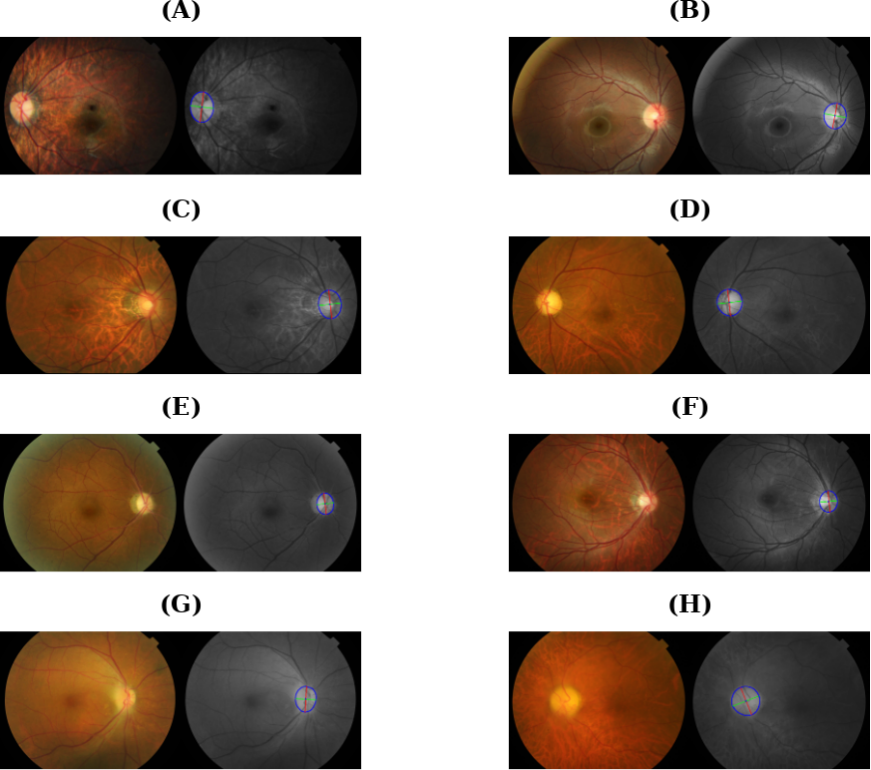
Distribution of tilt and nontilt classifications across disease types in the external validation dataset. Tilt classification was determined using the ratio of long diameter to short diameter, with a threshold of 1.3. (A) Normal tilt, (B) normal nontilt, (C) glaucoma tilt, (D) glaucoma nontilt, (E) pallor tilt, (F) pallor nontilt, (G) edema tilt, and (H) edema nontilt.

The performance of the proposed optic disc tilt classification pipeline was evaluated on the external SMC dataset. Figure 3 illustrates representative tilt and nontilt classifications across the 4 disease types, demonstrating that the LD and SD were stably computed based on the segmentation results. A quantitative summary of the tilt classification distribution by disease type is provided in Table 10. Of the 2448 images, 186 cases (7.5%) were classified as tilt and 2262 cases (92.5%) as nontilt. Edema had the highest tilt ratio (14/98, 14.2%), followed by normal (118/1215, 9.7%) and pallor (19/241, 7.8%), with glaucoma showing the lowest incidence (35/894, 3.9%).

To further investigate the association between myopia severity and optic disc tilt, we performed a subgroup analysis of patients with high myopia (spherical equivalent refraction ≤−6.0 D), as summarized in Table 11. Interestingly, even within this high-myopia subgroup, the glaucoma group exhibited a lower tilt prevalence (15/86, 17.4%) than the normal group (75/225, 33.3%). This discrepancy can be largely attributed to the substantial age difference between the cohorts. The normal group, with a mean age of 11.1 years, consisted primarily of children and adolescents likely undergoing progressive axial elongation, a process strongly associated with the development of optic disc tilt. In contrast, the glaucoma group (mean age 58.6, SD 12.8 years) may have included patients whose myopia originated from different etiologic pathways not necessarily involving disc tilting. Furthermore, potential selection bias cannot be excluded; diagnosing glaucoma in highly myopic eyes with tilted discs is notoriously challenging because of altered optic nerve head morphology, which may have led to an underrepresentation of such complex cases in our retrospectively collected cohort.

**Table 10.** Tilt classification results by disease type in the Samsung Medical Center external validation dataset.

Classification	Normal (n=1215)	Glaucoma (n=894)	Pallor (n=241)	Edema (n=98)	Total (N=2448)
Tilt, n (%)	118 (9.7)	35 (3.9)	19 (7.8)	14 (14.2)	186 (7.5)
Nontilt, n (%)	1097 (91.3)	859 (96.1)	222 (92.2)	84 (85.8)	2262 (92.5)

**Table 11.** Comparison of optic disc tilt prevalence between the overall cohort and the high-myopia subgroup.

Groups	Overall cohort	High-myopia group	Fold increase
Normal, n/N (%)	118/1215 (9.7)	75/225 (33.3)	3.43
Glaucoma, n/N (%)	35/894 (3.9)	15/86 (17.4)	4.46
Pallor, n/N (%)	19/241 (7.8)	4/17 (23.5)	3.02

### Segmentation Error Analysis on the SMC Dataset

**Figure 4.**
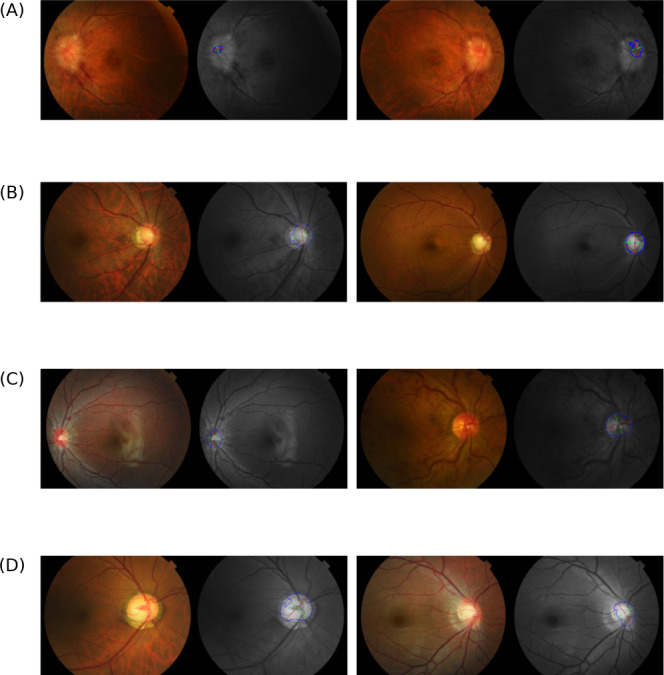
Optic disc segmentation error types. (A) Swelling-induced, (B) peripapillary atrophy–associated, (C) vessel boundary inference, and (D) other errors.

Despite the overall high accuracy, we conducted a granular error analysis to acknowledge the study’s limitations. Recognition errors occurred in 1.4% (34/2448; expert 1) and 1.14% (28/2448; expert 2) of the cases in the external validation dataset, and unacceptable cases identified by expert 1 were categorized into 4 types (Table 12; Figure 4): swelling-induced, peripapillary atrophy−related, vessel boundary interference, and other unclassified errors. Notably, a reduced clinical acceptance rate was observed in the edema group, where pathological swelling distorted disc boundaries (Figure 4A), causing the model to misidentify the disc extent. This segmentation unreliability may naturally compromise the accuracy of downstream tilt estimation.

**Table 12.** Summary of optic disc boundary recognition error types across disease types in the external validation dataset.

Error type^a^	Normal (n=1215), n (%)	Glaucoma (n=894), n (%)	Pallor (n=241), n (%)	Edema (n=98), n (%)	Total (n=2448), n (%)
Swelling-induced	0 (0)	0 (0)	0 (0)	15 (15.31)	15 (0.61)
Peripapillary atrophy–related	2 (0.16)	3 (0.33)	1 (0.04)	0 (0)	6 (0.25)
Vessel boundary interference	2 (0.16)	1 (0.11)	0 (0)	3 (3.06)	6 (0.25)
Other	2 (0.16)	5 (0.56)	0 (0)	0 (0)	7 (0.28)

a Error type categorization was based on cases flagged as clinically unacceptable by expert 1.

## Discussion

### Principal Findings

This study presents a robust AI-assisted pipeline for the automated detection and quantification of optic disc tilt, validated across diverse clinical conditions. By using an nnU-Net–based segmentation model trained on the SMDG dataset (3103 CFPs), we achieved outstanding performance that generalized effectively to the external SMC dataset (2448 CFPs), achieving mean clinical acceptance rates of 98.61% and 98.86% from 2 independent expert reviews. Our pipeline replaces subjective clinical judgments with an objective metric—the LD:SD ratio. Using a threshold of ≥1.3, the system identified 7.5% of images as tilted, with prevalence varying across the normal (9.7%), glaucoma (3.9%), pallor (7.8%), and edema (14.2%) groups. These findings affirm the model’s capacity to capture structural features across both healthy and diseased eyes, demonstrating robust boundary detection despite variations in image quality, race, and acquisition device.

The clinical implications of accurate optic disc tilt assessment are profound. Optic disc tilt occurs as a consequence of mechanical stress during axial elongation [14,43,44] and is correlated with the severity of myopic changes [4]. Tilted discs alter the 3D configuration of the optic nerve head, rendering traditional morphological criteria—such as the CDR and neuroretinal rim assessment—unreliable [6]. This structural distortion can mask genuine abnormalities or mimic pathological cupping, complicating the differentiation between physiological myopic changes and glaucoma [3]. Furthermore, tilt-induced alterations in retinal nerve fiber layer distribution can confound standard automated analyses [45,46]. By transitioning from manual, subjective assessments [47] to an automated, structure-based approach, our pipeline addresses these challenges. The integration of AI represents a paradigm shift in handling these geometric variations [48], overcoming the limitations of traditional algorithms that assume circular disc shapes [49]. The high agreement with expert judgment suggests that our system can reliably replicate human assessment [50] while reducing the interrater variability inherent in expert interpretations [51-53].

However, it is crucial to recognize that in clinical practice, the determination of optic disc tilt is of secondary importance in the presence of severe edema or papilledema. In such acute settings, the primary clinical objectives are the differential diagnosis of the underlying pathology and the preservation of vision rather than morphological profiling. Therefore, the inclusion of the edema group in this study was not intended to propose tilt assessment as a routine diagnostic step for these patients but rather to serve as a test of extreme conditions to evaluate the model’s robustness and boundary detection limits under challenging morphological conditions. Similarly, peripapillary atrophy regions mimicking disc intensity (Figure 4B) occasionally led to false boundary extensions. These findings highlight the inherent challenge of applying a model trained exclusively on public datasets to severe pathological cases. Future iterations could mitigate these errors through training with more diverse, disease-specific annotations, contributing to ongoing research on tilt classification [54,55]. In conclusion, this AI-assisted tilt detection system offers significant potential for enhancing clinical practice. It facilitates efficient, large-scale screening for myopia-related abnormalities and enables longitudinal monitoring of tilt progression. The objective quantification provided by this tool is particularly valuable for telemedicine applications, extending expert-level assessment to underserved regions [56]. Furthermore, tilt-aware diagnostics could reduce health care costs by preventing unnecessary treatments driven by false-positive findings [57]. Future research should focus on integrating multimodal imaging to further elucidate the pathophysiology of tilt-related complications. By providing a robust, reproducible solution to a complex diagnostic problem, this study represents a meaningful step toward precision medicine in ophthalmology.

### Conclusions

This study validated an AI-based pipeline for the automated detection and measurement of optic disc tilt on CFPs. By replacing manual segmentation and subjective clinical assessments, the pipeline provides objective and reliable tilt measurements, creating new possibilities for screening and diagnosing myopia-related eye conditions. The pipeline performed consistently across various clinical conditions (ie, normal, glaucoma, pallor, and edema) and effectively identified optic disc tilt in populations with a high prevalence of myopia. This system enhances the standardization and scalability of AI-driven diagnostics and shows potential for integration into telemedicine and large-scale screening programs. Future improvements, such as addressing challenges in edema cases and incorporating multimodal imaging, could further improve diagnostic accuracy. This work lays a strong foundation for improving the quality and accessibility of care for myopia-related eye diseases.

## Abbreviations

AI: artificial intelligence
CDR: cup-to-disc ratio
CFP: color fundus photograph
CNN: convolutional neural network
DSC: Dice similarity coefficient
LD: long diameter
SD: short diameter
SMC: Samsung Medical Center
SMDG: Standardized Multi-Channel Dataset for Glaucoma
